# Laboratory epidemiology of *Salmonella* infections and multi-drug resistance profiles in Nigeria: Barriers, challenges and proposed solutions

**DOI:** 10.1016/j.onehlt.2025.101289

**Published:** 2025-12-01

**Authors:** Amjad Banibella, Nooruldeen Saad, Jafar Eyad, Ramadhani Chambuso

**Affiliations:** a Pathological Sciences Department, Division of Microbiology, College of Medicine, Ajman University, United Arab Emirates; b Department of Global Health and Population, Harvard T. Chan School of Public Health, Harvard University, United States of America

**Keywords:** Laboratory epidemiology, ***Salmonella*** infections, Stool culture, Multi-drug resistance, Barriers and challenges

## Abstract

**Purpose:**

We investigated multi-laboratory culture records to quantify AMR profiles for *Salmonella* infections, diagnostic gaps and challenges in Nigeria.

**Methods:**

Using a retrospective study, we analyzed a total of 84,548 culture results from 26,630 patients across 25 public laboratories participated in the AMR surveillance report from Nigeria. *Salmonella* species and stool culture positivity rates were compared throughout the 3 year-period. Stool sampling gaps were quantified and *Salmonella* species AMR for key antibiotic classes were assessed. Chi-square test and Wald risk ratios were used for statistical analysis, a *p*-value of **<0.05** was considered statistically significant.

**Results:**

Out of 84,548 culture results, a total of 621 *Salmonella* species were isolated with *Salmonella Typhi* being the most commonly reported species. Stool samples represented only 3 % of all collected specimens, yet *Salmonella* species culture positivity increased from 64 % to 97 % (2016 to 2017; RR 1.51, 95 % CI 1.37–1.65, *p* < 0.001). AMR remained entrenched: trimethoprim–sulfamethoxazole ≥90%, fluoroquinolones ≥69 %, and nalidixic acid 91 %; cephalosporin resistance climbed from 60 % to 88 %. We identified a limited stool collection compared to other samples, which impacted identification of *Salmonella* infections in an endemic area like Nigeria. The key barriers were limited laboratory data integration and lack of One Health surveillance which amplified *Salmonella* infections AMR threat.

**Conclusion:**

Limited stool culture and escalating multi-drug resistance jeopardize the empirical therapy for *Salmonella* infections. Our study offers immediate, scalable interventions to strengthen One-Health *Salmonella* infections AMR control in Nigeria.

## Highlights

• Stool sampling for Salmonella was low but had the highest positivity rate.
• Resistance to fluoroquinolones and cephalosporins threatens *Salmonella* treatment.
• Limited species-level reporting on *Salmonella* cultures impacts AMR surveillance.
• Novel framework maps microbiological gaps to AMR control strategies for *Salmonella.*

## Graphical abstract

**Figure:**
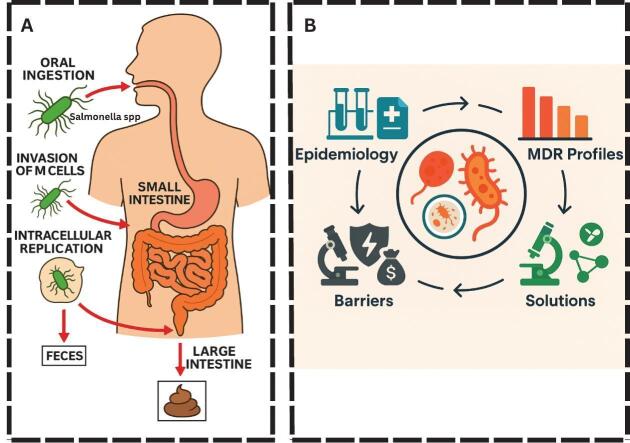

## 1 Introduction

*Salmonella* infections remain as the leading cause of foodborne illnesses globally, with 93.8 million cases of gastroenteritis and approximately 155,000 deaths each year, of which around 80 million cases (85 %) are attributed to contaminated food particularly poultry, eggs, pork, and dairy products. [[1], [2], [3], [4]] Invasive *Salmonella* infections present a particularly severe form of disease causing roughly 510,000 cases and emerging as a major public health concern in 2021, with the highest disease incidence and disability-adjusted life years (DALYs) concentrated in Sub-Saharan Africa and among infants under one year of age. [[5], [6], [7]] The rise of antimicrobial resistance (AMR) in *Salmonella* infections has become a critical global health concern, with a particularly severe health impact in endemic West African regions like Nigeria. [[8], [9], [10], [11]] Previous studies in *Salmonella* infections showed that the whole-genome sequencing has revealed a high prevalence of resistance genes. [[12], [13], [14], [15]] These resistance genes were among *Salmonella* isolates from poultry, with fluoroquinolone resistance nearing 70 % and widespread detection of extended-spectrum β-lactamase (ESBL) genes. [8,11,16,17]

Despite increasing reports of multidrug-resistant *Salmonella* infections in Nigeria, evidence remains limited on how the gaps in laboratory diagnostic practices can influence the accuracy and completeness of AMR detection and reporting. [[18], [19], [20], [21]] Specifically, microbiological gaps such as stool sample collection and culture for *Salmonella* infections, AMR testing protocols, and specific *Salmonella* infections policy alignment with the laboratory practices. [22] There is a critical need to identify and address the barriers and challenges to strengthen the national AMR surveillance and guide targeted interventions for *Salmonella* infections in different geographical areas. [[23], [24], [25]]

There are two primary species of concern: *Salmonella enterica* and *Salmonella bongori*, with *S. enterica* being responsible for most human infections. *S. enterica* is further classified into over 2500 serotypes, among which *Salmonella Typhi* is the most notable due to its role in causing typhoid fever, a severe systemic illness often found in areas with inadequate sanitation. [26,27] *Salmonella* is a genus of rod-shaped, Gram-negative bacteria that is a major cause of foodborne illnesses. [28] It belongs to the family *Enterobacteriaceae* and is primarily known for causing gastrointestinal infections, commonly referred to as salmonellosis. [29] *Salmonella* infections drug resistance to frontline treatments such as azithromycin has reached as high as 99.4 %, raising concern over treatment options for invasive infections. [30] This includes *Salmonella*'s ability to form biofilms, exchange resistance genes through horizontal gene transfer, and withstand environmental stresses. [31,32]

In this study, we investigated multi-laboratory culture records to quantify AMR profiles for *Salmonella* infections, microbiological gaps including stool culture, challenges and suggested for data-driven practical solutions.

## 2 Material and methods

### 2.1 Ethics statement

This study utilized publicly available, de-identified AMR surveillance data originally collected by the Nigerian Ministry of Health under the Fleming Fund Regional Grant (Phase 1): https://aslm.org/wp-content/uploads/2023/07/AMR_REPORT_NIGERIA.pdf?x89467 (accessed on 17 July 2025). No individual-level data were accessed. Secondary data use complied with the Declaration of Helsinki and did not require additional ethical clearance.

### 2.2 Study design

We conducted a retrospective study of the national AMR surveillance report of Nigeria for the years 2016 to 2018, similar to a published study. [33] This report, published in 2022, was generated through the Mapping Antimicrobial Resistance and Antimicrobial Use Partnership (MAAP) and involved 25 sentinel laboratories across Nigeria with bacteriology testing capacity. The study adheres to the STROBE (Strengthening the Reporting of Observational Studies in Epidemiology) guidelines.

### 2.3 Data sources and surveillance framework

The original data were collected by the Nigerian health authorities in collaboration with MAAP partners. Surveillance activities were carried out across a national network of medical laboratories selected for their bacteriology testing capacity. A total of 25 laboratories contributed antimicrobial susceptibility testing (AST) data using WHONET software, a standardized microbiology data management tool. Data collection involved trained field teams retrieving laboratory records from both paper-based and digital systems. Where feasible, these records were linked with hospital databases to include patient demographics and clinical metadata (e.g., age, sex, specimen type). This analysis uses routine culture and antimicrobial susceptibility data only, no genomic sequencing or molecular characterization was performed. Specimen collection followed clinical suspicion: blood culture for typhoid fever and systemic infections, stool culture for diarrhoeal illness or convalescent carriage.

### 2.4 Inclusion criteria and data management

Only *Salmonella* isolated with valid AST results were included in the analysis. In accordance with WHO Global Antimicrobial Resistance Surveillance System (GLASS) guidelines and Clinical and Laboratory Standards Institute (CLSI) M39-A4 recommendations, only the first isolate per patient per year was retained to avoid duplication. In cases where unique patient identifiers were not available, all isolates were retained and interpreted with appropriate caution. AST interpretations followed CLSI criteria, and where necessary, zone diameters or minimum inhibitory concentrations (MICs) were standardized using WHONET's interpretive rules to ensure consistency across laboratories. Because contributing laboratories differed in capacity, AST in the analyzed AMR report was performed by Kirby–Bauer disk diffusion at most sites and by automated MIC-based panels at some (the preparedness survey recorded automated AST in 9/73 **(**12.3 %) laboratories.

### 2.5 Data extraction from the report

In this study, we manually extracted relevant *Salmonella*-specific data from the national report, including; Total number of participated laboratories, collected specimens per year, source of collected specimens, specimen type, valid cultures, positive and negative cultures, species-level breakdown (*Salmonella Typhi*, *S. paratyphi*, *S. enterica*, and *Salmonella* spp.*)*, AST results for antibiotics tested and patient demographics (age and sex).

These variables were compiled into a structured Microsoft Excel dataset for convenient data analysis. Among the *Salmonella* isolates, specific species were identified whenever possible, while others were recorded generically as *Salmonella* spp. according to how they were recorded in the original report. AMR data were available for multiple antibiotics, allowing the analysis of year-wise AMR trends and identification of the most prevalent resistant antibiotic combinations.

### 2.6 AST and quality control

AST was conducted using both disk diffusion and minimum inhibitory concentration (MIC) panels, depending on the laboratory's capacity. For comparability, we analyzed S/I/R categories using common interpretive standards. The primary interpretive standard applied was the CLSI criteria. Quality control (QC) strains were widely used across labs for routine QC of AST procedures. External quality assessment (EQA) was implemented across participating laboratories via a structured proficiency testing program, coordinated with the support of MAAP and the Fleming Fund. Laboratories were encouraged to participate in periodic EQA rounds and corrective actions were documented for laboratories with poor performance. However, details on the proportion of laboratories consistently participating in EQA or specific inter-laboratory performance scores were not systematically reported.

### 2.7 AMR calculation

From the original report, AMR rates were derived from positive cultures with available AST results. AMR rates were calculated as the proportion of non-susceptible isolates (intermediate or resistant) relative to the total number of tested isolates within a single calendar year.

$$\mathrm{AMR}\;\mathrm{Rate}\left(\%\right)=\left(\frac{\mathrm{Number of non}-\mathrm{susceptible isolates}}{\begin{array}{c} \mathrm{Number of isolates tested for antimicrobial suscestibility} \end{array}}\right)x\;100$$

(95 % Confidence Interval)

Where:

• **AMR** = Antimicrobial Resistance
• *Non-susceptible isolates* = Resistant + Intermediate
• *Tested isolates* = All isolates subjected to AST

### 2.8 Statistical analysis

Statistical analyses were conducted using R version 4.3.2. Missing data were < 1 % and handled via complete-case analysis. We analyzed valid culture results using descriptive statistics to compare culture positivity across demographic groups and years. For stool samples, annual *Salmonella* positivity rates were calculated with 95 % Wald confidence intervals (CI), and pairwise risk ratios (RR) with 95 % CI were derived using 2016 as the reference year. The Wald log method was employed to estimate RRs, and heterogeneity across years was evaluated using χ^2^ tests. For categorical comparisons (e.g., sex, age group, year), proportions of positive versus negative cultures were assessed using Pearson's Chi-square test, with statistical significance defined as ***p* < 0.05**.

## 3 Results

### 3.1 Demographics of the total cultures and the characterization of the stool culture samples

Out of 84,548 valid cultures analyzed, 35.1 % of female samples yielded positive results compared to 28.3 % in males (***p* < 0.0001**). Age-stratified analysis revealed that positivity rates increased with age, peaking at 40 % in individuals older than 65 years, however, 42.5 % of all culture samples had unknown age. Over time, a significant upward trend in positivity was observed from 28.5 % in 2016 to 33.1 % in 2017 and 34.7 % in 2018 (***p* < 0.0001**), suggesting improved diagnostic yield. Importantly, AST was performed for over 95 % of positive cultures, ensuring robust downstream resistance profiling (Table 1).

**Table 1:** Demographics of the total cultures analyzed in the study population.

Variable	Group	Valid cultures, n	Positive cultures, n (%)	Negative cultures, n (%)	Positive cultures with AST, n	p-value*
Gender	Male	37,550	10,618 (28.3)	26,932 (71.7)	10,160	<0.0001
	Female	46,997	16,517 (35.1)	30,480 (64.9)	13,803	
Age	< 1 year	9689	2832 (29.2)	6857 (70.8)	2711	<0.0001
	1–17 (yrs)	20,476	4902 (23.9)	15,574 (76.1)	4526	
	18–49 (yrs)	29,336	9323 (31.8)	20,013 (68.2)	7629	
	50–65 (yrs)	5177	1753 (33.9)	3424 (66.1)	1630	
	Above 65 (yrs)	4743	1898 (40.0)	2845 (60.0)	1806	
	Unknown age	15,127	6427 (42.5)	8700 (57.5)	5661	
Year	2016	26,896	7678 (28.5)	19,218 (71.5)	6532	<0.0001
	2017	32,134	10,640 (33.1)	21,494 (66.9)	9501	
	2018	25,518	8817 (34.6)	16,701 (65.4)	7930	

*Pearson χ^2^ test comparing the proportion of positive versus negative cultures across all levels of each variable. Total number of cultures, (*N* = 84,548).

In order to quantify the annual *Salmonella* positivity rates in stool cultures and assess the temporal trends in *Salmonella* detection from stool samples, we analyzed whether laboratory yield improved over time and if outbreak signals were emerging, or if diagnostic protocols required were strengthening. We are revealing a substantial and statistically significant rise in *Salmonella* infections positivity rate from 64.2 % in 2016 to over 89 % in 2018 (39 % higher, ***p* < 0.001**, Table 2).

**Table 2:** Characterization of stool samples for each year and the Salmonella yield.

Year	Stool samples (n)	Salmonella-positive (n)	Positivity % (95 % CI)	Pairwise Risk ratio vs 2016 (95 % CI) ⁎	p-value⁎⁎
2016	254	163	64.2 % (58.1–69.8)	Reference	< 0.001
2017	266	257	96.6 % (93.7–98.2)	1.51 (1.37–1.65)	
2018	225	201	89.3 % (84.6–92.7)	1.39 (1.26–1.54)	
Total	745	621	83.4 % (80.5–86.0)	n/a	

⁎ Pair-wise risk ratios (RR) were calculated with 95 % CIs using Wald log-method.

⁎⁎ Overall heterogeneity in positivity across years: χ^2^ = 106.9, *P* < 0.001 (2 d.f.).

### 3.2 Collected specimens' distribution, culture positivity rate, and ***Salmonella spp profile***

**Fig. 1:**
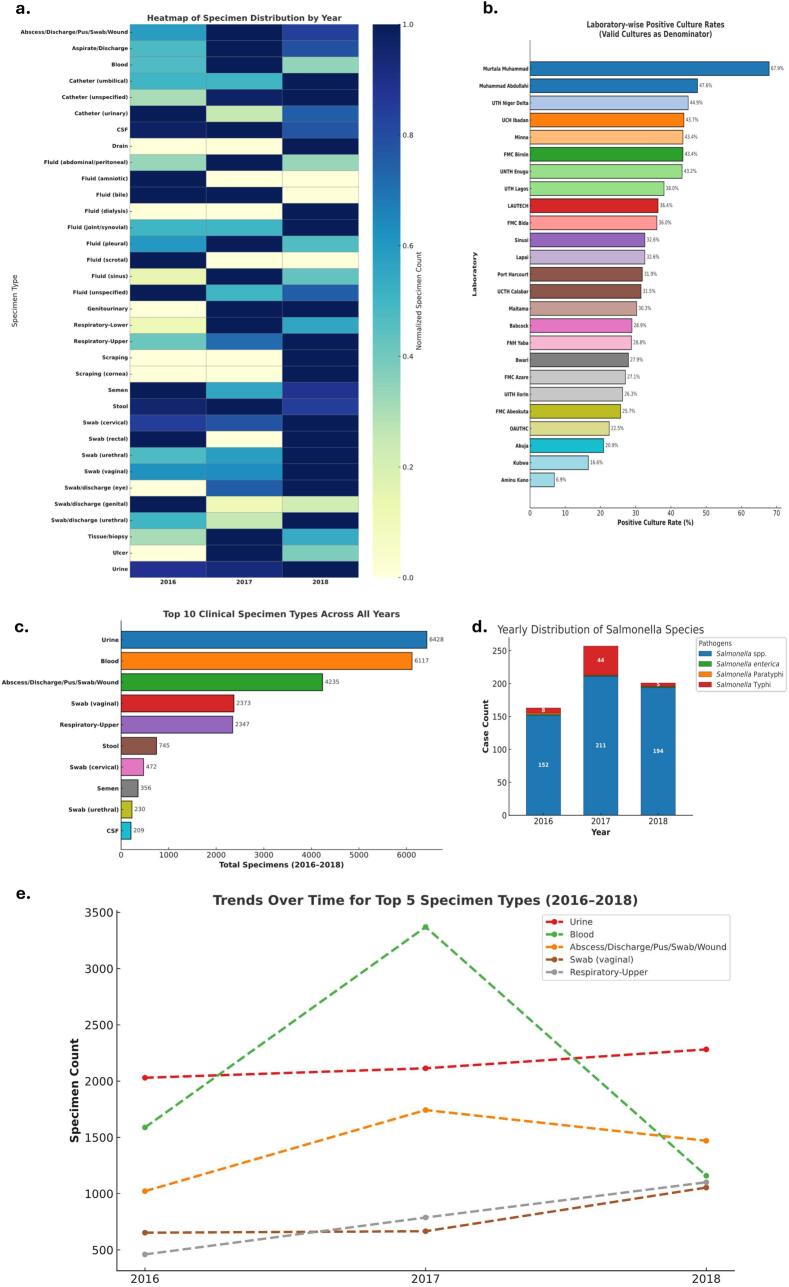
**Patterns of specimen distribution, culture positivity, and *Salmonella spp profile*.** (**a**) Distribution of specimen types by year showing all specimens collected. (**b**) Laboratory-wise culture positivity showing wide variability (6.9 %–67.5 %. (**c**) Top ten clinical specimen types collected, showing stool samples were the sixth most collected (**d**) Yearly *Salmonella* species distribution shows a notable spike in *S. typhi* in 2017. (**e**) Trend analysis of the five most collected specimen types reveals a sharp rise and fall in blood sample collection in 2017 whereas stool remained excluded from leading collected specimen categories throughout.

Further analyses of collected specimen patterns and laboratory performance revealed critical microbiological insights for *Salmonella* surveillance. Fig. 1a shows that while stool specimens were consistently submitted across all years, other diagnostically relevant fluids such as bile and peritoneal aspirates remained underutilized, suggesting sampling blind spots in cases of invasive *Salmonella* infections. In Fig. 1b, laboratory-level culture positivity ranged widely from 6.9 % to 67.5 %, indicating stark disparities in diagnostic performance that may compromise pathogen recovery, particularly in under-resourced sites. Despite high *Salmonella* yield, stool samples ranked only sixth among all specimen types (Fig. 1c), reinforcing the disconnect between clinical practice and the need for stool-based diagnostics. Fig. 1d identifies a sharp rise in *S.* typhi positive cultures in 2017 (*n* = 44), consistent with a potential outbreak, although species-level resolution for *S. enterica* and serotype-level resolution for *S. paratyphi* remained limited. Blood specimen submissions peaked in 2017 (Fig. 1e**)**, coinciding with the rise in typhoid cases in the same year, however, stool cultures did not appear among the top five specimen types, further underscoring diagnostic underutilization. Across 2016–2018, stool accounted for 745 submissions and did not feature among the top five specimen types in any year (Fig. 1c–e), whereas blood and urine dominated volumes. This pattern indicates selective, indication-based stool sampling rather than routine screening. Collectively, these findings point to critical gaps in laboratory practices, specimen prioritization, and species-level identification that must be addressed to enhance *Salmonella* detection and response across endemic regions, like Nigeria. [34]

### 3.3 AMR surveillance in all clinical isolates and the culture for *Salmonella* species

**Fig. 2:**
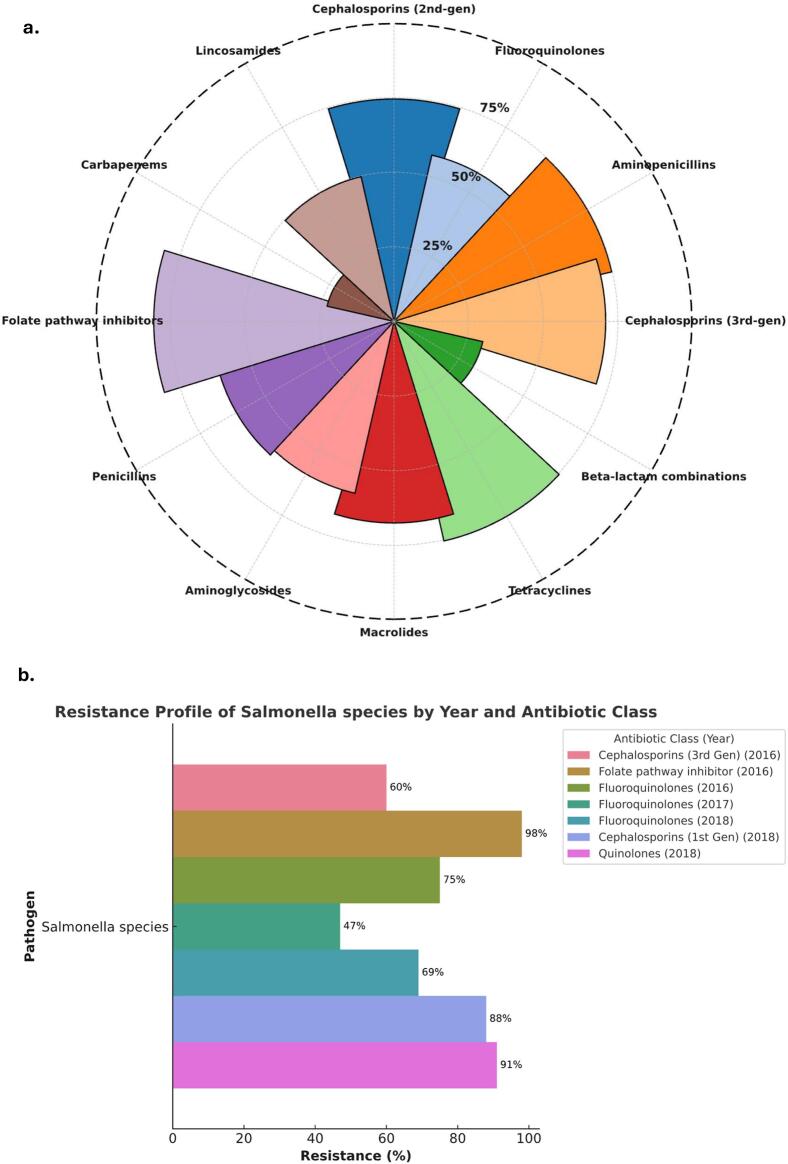
**AMR surveillance in all clinical isolates and *Salmonella* Species**. (**a**) Presents the overall antimicrobial resistance profile across all clinical pathogens in the dataset, revealing highest resistance rates against folate pathway inhibitors, aminopenicillins, and third-generation cephalosporins. (**b**) Shows specifically on *Salmonella* species on the marked temporal rise in resistance: fluoroquinolone resistance increased from 47 % in 2017 to 69 % in 2018.

The AMR landscape showed a deepening crisis in the treatment of enteric infections. The overall resistance burden across the full clinical dataset shows that several key antibiotic classes such as aminopenicillins, third-generation cephalosporins, and folate pathway inhibitors exhibited resistance rates exceeding 70 % (Fig. 2a). This cross-pathogen resistance profile points to intense selection pressure and uncontained antimicrobial misuse within the system. In contrast, when we focused specifically on *Salmonella* species AMR, we uncovered a worrying temporal escalation. Between 2016 and 2018, resistance to fluoroquinolones rose sharply from 47 % to 69 %, while quinolone and first-generation cephalosporin resistance reached 91 % and 88 %, respectively, by 2018 (Fig. 2b). Notably, even in 2016, *Salmonella* isolates already showed near-complete resistance to folate pathway inhibitors (98 %) and concerning levels to third-generation cephalosporins (60 %) (Fig. 2b).

### 3.4 Critical microbiological and surveillance gaps undermining effective *Salmonella* AMR control

**Fig. 3:**
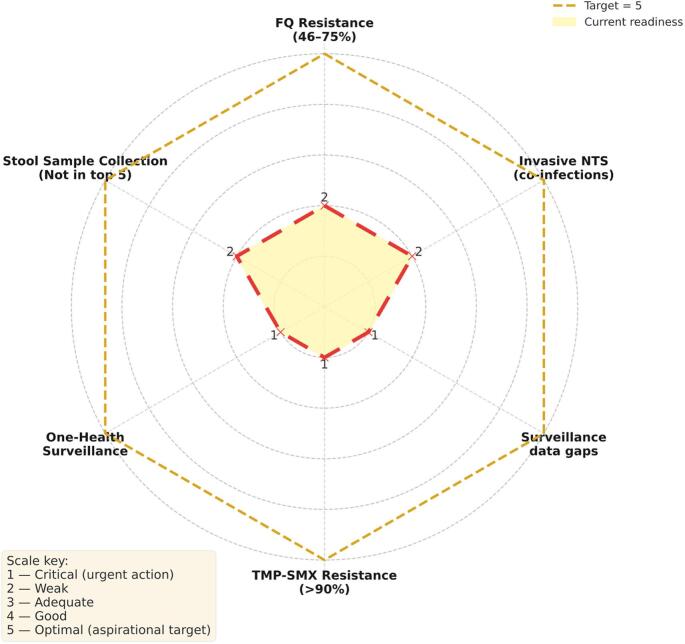
***Salmonella* AMR gaps-readiness analysis (1–5 scale). There are** six priority domains**:** FQ resistance (46–75 %)**,** invasive NTS (co-infections)**,** surveillance data gaps**,** TMP-SMX resistance (>90 %)**,** One-Health surveillance, and **stool sample collection (not in top-5 by volume)**. The **shaded polygon with dashed outline** shows the **current readiness score** for each domain **(**1 = critical deficit; 5 = optimal); the **dashed outer ring** marks the **aspirational target (5)**. Numeric labels at each vertex indicate the assigned score. Axis parentheticals provide contextual statistics from the dataset; the figure is a qualitative synthesis to guide prioritization. *Abbreviations:***FQ** = fluoroquinolone; **TMP-SMX** = trimethoprim-sulfamethoxazole; **iNTS** = invasive non-typhoidal *Salmonella*.

To better contextualize the critical microbiological challenges surrounding *Salmonella* spp. AMR, we summarized key microbiological and surveillance gaps identified. This synthesis analyzed six interlinked gaps that, taken together, impede effective detection, treatment, and containment of drug-resistant *Salmonella* strains. These gaps span between both microbiological and system-level domains, including resistance to key antibiotic classes, under-prioritization of stool sampling, and lack of integrated One Health surveillance (Fig. 3). [35] We highlighted what remains neglected in *Salmonella* AMR response efforts across clinical and public health settings. The observed gaps include:

(i) **Fluoroquinolone (FQ) resistance (46–75 %)**
  Resistance to fluoroquinolones span from 46 % to 75 %, indicating that nearly half to three-quarters of *Salmonella* isolates tested were non-susceptible to this drug class.
(ii) **Invasive non-Typhoidal *Salmonella* (iNTS) in co-Infections**
  A distinct category flags invasive NTS associated with co-infective presentations. This was recorded as a discrete surveillance element, separating iNTS through bloodstream diagnosis from the broader pool of gastrointestinal cases. [36]
(iii) **Surveillance data gaps**
  There was observed surveillance data gap confirming incomplete or missing laboratory information, specifically, deficiencies in detailed isolate characterization (e.g., species, serovar, and susceptibility metadata).
(iv) **Trimethoprim–Sulfamethoxazole (TMP-SMX) resistance (> 90 %)**
  Resistance to TMP-SMX surpasses the 90 % threshold, signifying that fewer than one in ten *Salmonella* isolates remained susceptible to this long-standing drug.
(v) **Absence of integrated One-Health surveillance**
  The “One-Health surveillance” was recorded as a gap, denoting that coordinated data capture across human, animal, and environmental sources was insufficiently documented for *Salmonella*.
(vi) **Stool sample collection for *Salmonella* culture** and AST
  Stool sampling was explicitly not in top 5 most collected samples for culture, confirming that, despite its relevance for enteric pathogens, stool was collected less frequently than at least five other specimen categories across the study years (Fig. 3).

### 3.5 Microbiological recommendations for strengthening *Salmonella* AMR control

**Fig. 4:**
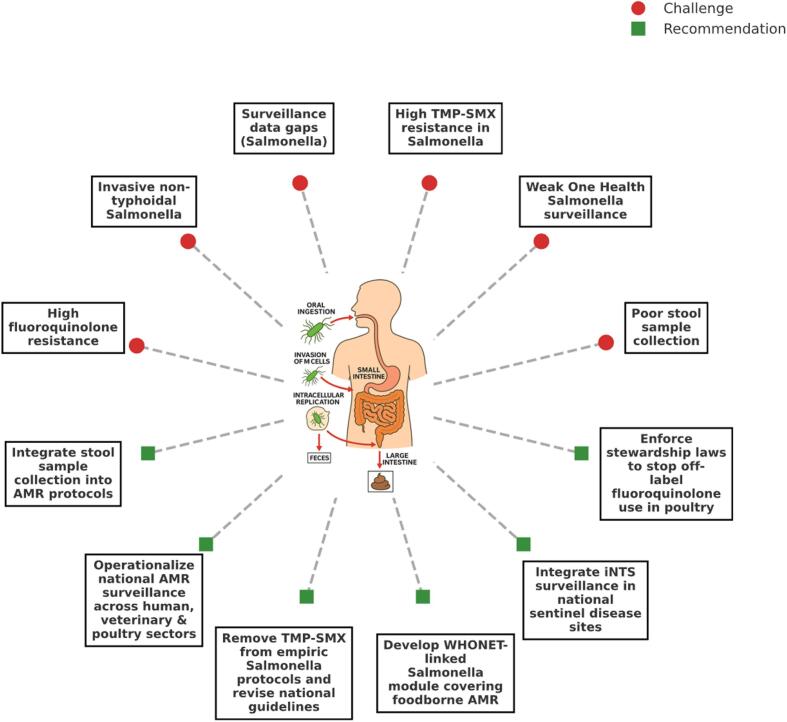
**Microbiological challenges and strategic recommendations to strengthen *Salmonella* AMR control**. This conceptual framework analyzed form the data to illustrate potential nine microbiological and surveillance challenges surrounding *Salmonella* antimicrobial resistance (red circles), each linked to targeted, actionable recommendations (green squares). This synthesis bridges clinical microbiology, AMR policy, and pathogen ecology, offering a roadmap for strengthening diagnostic prioritization and containment strategies in endemic settings. (For interpretation of the references to colour in this figure legend, the reader is referred to the web version of this article.)

To translate the observed microbiological gaps into actionable priorities, a targeted framework of laboratory-informed recommendations was developed (Fig. 4**)**. This approach systematically maps each challenge ranging from AMR and underdiagnosis to surveillance fragmentation against practical interventions rooted in microbiological best practices. These recommendations aim to improve *Salmonella* spp. detection, guide treatment decisions, and enhance cross-sectoral AMR surveillance capacity, particularly in high-burden and resource-limited settings. The recommendations are as follows:

(i) **Integrate stool sample collection into AMR protocols**

Routine stool sampling must be embedded into national AMR surveillance protocols, particularly for suspected *Salmonella* infections. Stool specimen is essential for identifying gastrointestinal *Salmonella* strains, yet underutilization, previously demonstrated by its absence from the top five specimen types, undermines pathogen recovery. Integration into diagnostic algorithms will improve case detection, enable species-level typing, and ensure more representative resistance profiling. Stool culture is appropriate for gastroenteritis/NTS and for carriage assessment; it is not the standalone diagnostic for invasive typhoid, where blood culture is indicated. So, our recommendation still priorities blood culture for suspected enteric fever and situate stool culture within syndromic algorithms. [37,38]

(ii) **Enforce stewardship Laws to stop off-label fluoroquinolone use in poultry**
  Strengthen regulatory oversight and surveillance of veterinary antimicrobial use, particularly fluoroquinolones, given documented FQ-resistant *Salmonella* in Nigerian poultry and AMR associations. [[39], [40], [41]] Enforcing antimicrobial stewardship regulations targeting veterinary sectors is crucial to reduce selection pressure and prevent further resistance amplification in zoonotic reservoirs.

(iii) **Remove TMP-SMX from empiric *Salmonella* protocols and revise national guideline**

Given the AMR of >90 % observed for TMP-SMX, national treatment guidelines should be updated to exclude it from empirical therapy for suspected *Salmonella* cases. This change is critical to prevent ineffective treatment, mitigate resistance escalation, and redirect clinicians toward evidence-based, susceptibility-guided *Salmonella* treatment. [42]

(iv) **Operationalize national AMR surveillance across human, veterinary, and poultry sectors**

To capture the full epidemiological context of *Salmonella* transmission, surveillance must be extended beyond clinical settings to include veterinary and poultry sectors. This recommendation aligns with a One Health framework, ensuring coordinated sampling, data integration, and resistance monitoring across reservoirs that perpetuate resistant *Salmonella* strains. [18]

(v) **Develop WHONET-linked *Salmonella* module covering foodborne diseases AMR**

A dedicated *Salmonella* module within the WHONET platform would enable centralized, standardized tracking of resistance trends in foodborne disease isolates. This module would allow microbiologists to monitor susceptibility shifts in real time, detect emerging resistance phenotypes, and correlate food source data with clinical outcomes. [43]

(vi) **Integrate iNTS surveillance in national sentinel disease sites**

iNTS infections often associated with bloodstream infections in immunocompromised individuals require dedicated monitoring within sentinel sites. Integration of iNTS surveillance into national systems will enable early detection, facilitate targeted intervention, and support the development of diagnostics and vaccines specific to this under-recognized but clinically significant form of *Salmonella* infections (Fig. 4**)**. [36]

## 4 Discussion

We analyzed a total of 84,548 valid clinical cultures from Nigeria collected from 2016 to 2018. The specimen isolates distribution for *Salmonella* infections, detection patterns, AMR profiles, and surveillance gaps were systematically analyzed across multiple diagnostic laboratories. We revealed several novel insights: despite the highest *Salmonella* culture positivity, stool samples remained significantly underutilized; species-level identification was largely absent or underreported; and fluoroquinolone resistance in *Salmonella* rose sharply, surpassing 69 % by 2018. We also identified significant inter-laboratory variation in culture positivity (6.9 %–67.5 %) and extremely high TMP-SMX resistance (>90 %), both previously underreported in regional AMR surveillance. Importantly, we developed a novel microbiological-driven gap analysis and recommendations framework linking diagnostic gaps to targeted interventions, including WHONET integration, empirical therapy revision, and One Health surveillance. [44] Consistent with clinical guidance, blood culture is the mainstay for suspected enteric fever, while stool testing is reserved for diarrhoeal illness and for carriage assessment**.** Stool yield for *S. typhi*/Paratyphi is lower and can reflect convalescence/carriage. Moreover, multiple-pathogen results (particularly with multiplex assays) require clinical correlation because detection of nucleic acid does not always indicate viable, etiologic infection. [37,38]

Although our data were derived from human clinical laboratories, the recommendations integrate with GLASS and Africa CDC guidance for One Health AMR surveillance with standardized data structures, harmonized AST panels/QC and cross-sector reporting that links human clinical signals to animal and environmental streams. This framing converts descriptive surveillance into system improvement: a practical path from laboratory metrics to coordinated action across sectors in One Health. This translational approach offers a scalable model for enhancing clinical microbiology for *Salmonella* control and AMR containment in low-resource, high-burden settings like Nigeria. [42,45,46] Our dataset reflects surveillance between 2016 and 2018 which offers a critical baseline for tracking Nigeria's AMR response trajectory and for benchmarking future diagnostic improvements within the Fleming Fund and GLASS frameworks. These findings suggest a critical disruption in the microbiological ecosystem of *Salmonella* spp., where diagnostic underperformance and limited AMR evolution are converging. The consistent lack of species-level identification hampers the understanding of circulating *Salmonella* lineages, masking shifts in virulence and resistance phenotypes that are essential for guiding outbreak responses, vaccine targeting, and phage therapy design. In addition, the rise of multidrug-resistant *Salmonella* compromises the utility of both oral and parenteral treatment regimens. This may narrow therapeutic windows and increase reliance on last-resort agents. This reflects the limited spread of mobile *Salmonella* genetic elements and clonal expansion of resistant strains, which, if unaddressed, could establish persistent reservoirs across clinical, foodborne, and environmental niches. Recent genomic syntheses demonstrate human-to-human transmission in iNTS and lineage-specific AMR expansions in *S. typhi* (e.g., H58), while new dashboards such as TyphiNET operationalize Whole Genome Sequencing for public-health action. Our findings therefore sit within a landscape where serovar-resolved surveillance is increasingly critical for control. [14,[47], [48], [49]]

Previous literature on *Salmonella* infections in Nigeria has largely focused on outbreak descriptions, bloodstream infections, or molecular typing in isolated settings, with limited integration of diagnostic workflow data, stool specimen utilization, and real-time AMR patterns. [[50], [51], [52], [53]] Our study bridges that gap by revealing the operational disconnect between high-yield diagnostics such as stool cultures and their actual underutilization in routine practice. While previous reports have documented rising fluoroquinolone resistance, our study provides robust temporal and cross-laboratory evidence of its progression, supported by context-specific resistance thresholds across antibiotic classes. [54] Furthermore, the striking absence of species-level *Salmonella* identification aligns with known challenges in LMIC microbiology but has rarely been quantified across systems. Of 621 *Salmonella*-positive cultures, 64 (10.3 %) had a serovar/species recorded (Typhi 57, Paratyphi 4, *S. enterica* 3), while 557 (89.7 %) were reported as *Salmonella* species. This marked increase in positivity from 64 % (2016) to 89 % (2018) may reflect improved laboratory capacity, enhanced participation in external quality assurance and increased availability of diagnostic reagents supported by the Fleming Fund Phase 1 activities. However, there is also a possibility of a true rise in incidence since Nigeria is an endemic area for *Salmonella* infection. Improving attribution is a surveillance priority consistent with GLASS metadata standards and WHONET implementation guidance. [37] This work adds value by providing the first structured linkage between microbiological failure points and practical, lab-driven interventions, thereby elevating the role of microbiologists from passive reporters to active architects of AMR response and surveillance reform.

We exposed hidden microbiological failures in *Salmonella* infection detection and resistance containment gaps that traditional surveillance studies often overlook. By triangulating specimen practices, pathogen recovery rates, and *Salmonella*-specific resistance data, we reveal how stool under-sampling, poor species resolution, and inconsistent laboratory performance collectively distort the microbiological landscape of enteric fever caused by *Salmonella* infections. [55] Unlike previous studies limited to single-centre or outbreak reports, this work unifies fragmented data into an actionable framework, highlighting the structural and diagnostic blind spots that perpetuate *Salmonella* misdiagnosis and AMR progression. [56] This integrative approach provides microbiologists with a blueprint for reforming diagnostic priorities and surveillance architecture in *Salmonella* endemic regions.

We translated microbiological data into pragmatic interventions that are both context-sensitive and scalable. By aligning laboratory findings with system-level diagnostics and policy gaps, our gap analysis study enables targeted improvements such as integrating stool sampling into AMR protocols, eliminating ineffective antibiotics from empirical regimens, and operationalizing One Health surveillance platforms. Our recommendations are practical and data oriented, derived directly from field-level diagnostic perspectives, ensuring feasibility and translational relevance. Importantly, the new framework proposed in this study serves not only as a diagnostic audit tool but also as a decision-making scaffold for health ministries, AMR committees, and laboratory networks seeking to enhance *Salmonella* control through sustainable microbiology-led interventions for resource-limited countries. [11,57]

**Key strengths of this study include:**

(i) **First to link diagnostic performance with *Salmonella* treatment gaps** by establishing a unique correlation between underdiagnosis, resistance evolution, and policy inaction in endemic contexts.
(ii) **Large multi-year, multi-site dataset from *Salmonella* infection endemic region.** Analysis of 84,548 valid cultures across multiple diagnostic centres over three years provides robust, generalizable findings.
(iii) **Microbiologically-focused gap analysis** study which identified a novel framework for diagnostic and surveillance blind spots specific to *Salmonella infections*, including stool specimen prioritization, species identification, and AMR trends.
(iv) **Time-trend AMR profiling** which showed the escalating *Salmonella* resistance to fluoroquinolones and cephalosporins in a temporal, multi-centre context from a LMIC setting.
(v) **Actionable laboratory-based recommendations that** directly translated findings into implementable solutions such as empirical treatment revisions, WHONET integration, and One Health coordination in real world.
(vi) **New systems-level diagnostic insights** which integrated specimen workflow data with microbiological outcomes specific for *Salmonella* infections, exposing variability in stool culture positivity and diagnostic capacity for each year.
(vii) **Translational relevance to public health** by providing microbiological evidence to inform *Salmonella* infections AMR combating strategies, health ministries, and laboratory networks seeking to enhance *Salmonella* infections surveillance and treatment policies in limited-resource countries.
(viii) **Scalable and reproducible methodology** which offers a replicable model for other LMICs to evaluate microbiology systems and align laboratory practices with global AMR control strategies for *Salmonella* infections.

Limitations include;

(i) AMR profiles were inferred from phenotypic data without confirmatory genotypic or plasmid-based analysis.
(ii) Most *Salmonella* isolates were not identified to the species or serovar level, restricting lineage-specific interpretations and outbreak mapping.
(iii) Missing patient-level clinical data (e.g., HIV status, antibiotic exposure, comorbidities) limited the ability to link resistance with clinical outcomes.
(iv) Inter-laboratory differences in methods and quality assurance may have influenced *Salmonella* culture positivity and AMR reporting, introducing bias.
(v) Despite identifying One Health gaps, the study lacked animal or environmental sources, limiting source attribution.
(vi) The use of secondary data and the findings may not fully reflect recent national trends in Nigeria, especially in rural or informal health settings not represented in the dataset or recent years. Nevertheless, the dataset provides an essential baseline for longitudinal system evaluation and AMR capacity benchmarking.
(vii) Potential underreporting of negative cultures could skew specimen trends for *Salmonella*.
(viii) AMR dataset we analyzed does not include clinical working diagnoses, so we could not stratify stool cultures by indication.

Despite these limitations, our study provides valuable microbiology-focused analysis of *Salmonella* infections diagnostics and resistance trends in a West-African context. Its strength lies in transforming routine laboratory data into actionable insights and revealing systemic microbiological blind spots previously unreported. The translational framework and scalable recommendations make it a timely and impactful contribution to global AMR response efforts in new microbiological perspectives.

## 5 Conclusions

This study successfully identified critical weaknesses in stool specimen collection, species-level identification for *Salmonella* infections, and AST that compromise the detection, reporting, and control of *Salmonella* infections in endemic settings. For *Salmonella* infections, enhancing stool culture prioritization, promoting species-level identification, and integrating real-time resistance surveillance (e.g., WHONET-linked modules) are urgent and feasible actions. By linking these diagnostic and surveillance failures with escalating resistance to first-line and second-line antibiotics, our study addresses a major gap in contextualizing laboratory performance within the broader AMR crisis for *Salmonella* infections. Future work should build on this framework to expand One Health AMR monitoring, incorporate molecular diagnostics, and strengthen laboratory networks to translate microbiological data into early warning and containment systems for *Salmonella infections* and other enteric pathogens.

The following are the supplementary data related to this article.

Supplementary Table 1 Phased build-out of Salmonella serotyping, molecular confirmation and santinel whole genome sequensing. Supplementary Table 1

Supplementary Table 2 Standards-aligned, measurable actions and KPIs derived from national Salmonella surveillance findings. Supplementary Table 2

Supplementary material 1: Dataset used for analysis Image 1

Supplementary material 2: STROBE Statement-checklist of items that should be included in reports of observational studies. Image 2

## CRediT authorship contribution statement

**Amjad Banibella:** Writing – review & editing, Writing – original draft, Validation, Methodology, Data curation, Conceptualization. **Nooruldeen Saad:** Writing – review & editing, Writing – original draft, Validation, Methodology, Investigation, Data curation. **Jafar Eyad:** Writing – review & editing, Writing – original draft, Validation, Methodology, Investigation, Data curation. **Ramadhani Chambuso:** Writing – review & editing, Writing – original draft, Visualization, Validation, Supervision, Software, Resources, Project administration, Methodology, Investigation, Formal analysis.

## Declaration of generative AI and AI-assisted technologies in the writing process

During the preparation of this work the authors used *ChatGPT5 (OpenAI, San Francisco, USA)* to improve readability and language. After using this tool/service, the authors reviewed and edited the content as needed and take full responsibility for the content of the published article.

## Funding source

This research did not receive any specific grant from funding agencies in the public, commercial, or not-for-profit sectors.

## Declaration of competing interest

The authors declare that they have no known competing financial interests or personal relationships that could have appeared to influence the work reported in this paper.

## Data Availability

Data used in this research is available in supplementary material.
