# Supplementary material for: Laboratory epidemiology of Salmonella infections and multi-drug resistance profiles in Nigeria: Barriers, challenges and proposed solutions
Source: One Health. 2025 Dec 1;21:101289. doi: 10.1016/j.onehlt.2025.101289 (PMC12719698; doi:10.1016/j.onehlt.2025.101289)
Supplement: Supplementary Table 1 — Phased build-out of Salmonella serotyping, molecular confirmation and santinel whole genome sequensing. [file mmc1.docx]

**Supplementary Table S1.** Phased build-out of *Salmonella* serotyping, molecular confirmation and sentinel whole genome sequencing

| **Phase & Timeline** | **Laboratory tier** | **Purpose** | **Core method(s) & minimal reagents** | **SOP / QC / EQA** | **Data capture & integration** | **Primary outputs** | **1st‑year KPIs** | **References** |
| --- | --- | --- | --- | --- | --- | --- | --- | --- |
| Phase 1 (0–6 months) | Sentinel labs; confirm atypical results at National Reference Lab (NRL) | Rapid, low‑cost serotyping to distinguish Typhi/Paratyphi and major O groups; establish SOP/QC baseline | Slide‑agglutination per Kauffmann–White–Le Minor; minimal antisera panel: Poly‑O (A–G, A–S), Poly‑H, O:2, O:4, O:9, Vi | ISO/TR 6579‑3; daily positive/negative controls; quarterly EQA | WHONET fields: species, serovar (Typhi/Paratyphi A/B/C/other), O/H factors; flag “atypical sent to NRL” | Typhi vs Paratyphi assignment for most isolates; improved serovar‑stratified antibiograms | ≥85% of *Salmonella* isolates serotyped; 100% atypical referred to NRL; ≥95% records complete | [1], [2] |
| Phase 2 (6–12 months) | Hub labs / NRL | Resolve antisera‑unavailable/ambiguous cases; screen high‑risk lineages; enable low‑infrastructure confirmation | Targeted molecular assays: multiplex PCR (Typhi vs Paratyphi A); optional LAMP (locally validated) for Salmonella targets; add H58/XDR markers where available | Extraction controls, positive/negative PCR controls, inhibition checks; verify against serotyping where possible | Add molecular result fields in WHONET/LIS (Typhi/Paratyphi PCR, lineage markers); link to specimen metadata | Serotype confirmation when antisera limited; earlier flagging of high‑risk clones; reduced turnaround time | ≥90% concordance PCR vs serotyping; ≥80% of unresolved isolates tested; median TAT ≤ 24 h post‑culture | [3] |
| Phase 3 (12–24 months) | NRL (hub‑and‑spoke submissions) | Lineage‑level AMR surveillance, outbreak linkage, cross‑border comparability | Sentinel whole‑genome sequencing (quarterly panel of epidemiologically selected isolates); analyze via Pathogenwatch/Typhi Genomics; contribute to TyphiNET | Library/QC standards (coverage ≥30×, contamination checks); external WGS proficiency testing | Export lineage/AMR calls back to WHONET/LIS; issue public‑health genomic reports | Validated AMR genotype–phenotype concordance; cluster detection; regional lineage maps | Sequence ≥10–20% of national Salmonella Typhi/Paratyphi each quarter; ≤ 60 days from collection to report; 100% uploads to Pathogenwatch/TyphiNET | [4], [5] |

**References**

1. ISO/TR 6579‑3:2014. Microbiology of the food chain—Guidelines for serotyping of Salmonella (serotyping SOP/QC).

<https://cdn.standards.iteh.ai/samples/56712/37da386eff674e07b35f9025371ee283/ISO-6579-1-2017.pdf>

1. Thermo Fisher Scientific. Salmonella Agglutinating Sera (Kauffmann–White–Le Minor antisera panel) —

Manual X7823A. <https://tools.thermofisher.com/content/sfs/manuals/X9423.pdf>

3. Fabre L, et al. CRISPR‑targeted PCR for identification of S. Typhi and S. Paratyphi A. PLoS Negl Trop Dis.

2014;8(1):e2671. <https://doi.org/10.1371/journal.pntd.0002671>

1. Argimón S, et al. Pathogenwatch for S. Typhi AMR prediction and genomic context. Nature

Communications. 2021;12:2879. <https://doi.org/10.1038/s41467-021-23091-2>

1. Global Typhoid Genomics Consortium. TyphiNET dashboard for public‑health genomic reporting. Genome

Medicine. 2025;17(1):51. <https://doi.org/10.1186/s13073-025-01470-4>
