# Supplementary material for: Laboratory epidemiology of Salmonella infections and multi-drug resistance profiles in Nigeria: Barriers, challenges and proposed solutions
Source: One Health. 2025 Dec 1;21:101289. doi: 10.1016/j.onehlt.2025.101289 (PMC12719698; doi:10.1016/j.onehlt.2025.101289)
Supplement: Supplementary Table 2 — Standards-aligned, measurable actions and KPIs derived from national Salmonella surveillance findings. [file mmc2.docx]

**Supplementary Table S2.** Standards‑aligned, measurable actions and KPIs derived from national *Salmonella* surveillance findings

| **No.** | **Action (what to implement)** | **Metric / KPI (definition) & 12‑month target** | **Reporting cadence & owner** | **Rationale from our data** | **Refs** |
| --- | --- | --- | --- | --- | --- |
| 1 | Specimen‑yield stewardship dashboard across sites for blood, stool, bile/peritoneal (publish monthly submission: yield indices; set site targets). | KPI‑1: % sites publishing monthly dashboard (target ≥95%). KPI‑2: CV of specimen yields across sites reduced ≥20% vs baseline. KPI‑3: Stool submissions per 100 diarrhoeal requests increase vs baseline. | Monthly; site microbiology leads; oversight by the National Reference Laboratory (NRL) AMR team. | Stool under‑submission; need to balance specimen mix for interpretable AMR. | [1] |
| 2 | Serovar completeness KPI: add Typhi/Paratyphi/iNTS fields to national WHONET schema; report % isolates with serovar quarterly. | KPI‑1: Serovar field completeness ≥85% at 6 mo and ≥95% at 12 mo. KPI‑2: Quarterly serovar distribution report published. | Quarterly, NRL WHONET administrator. | Serovar completeness ~10.3% at baseline limits epidemiology and stewardship. | [2] |
| 3 | CLSI M39‑compliant antibiogram displays: enforce first‑isolate rules, minimum n, and deduplicated outputs; report AST‑coverage rate. | KPI‑1: ≥90% labs apply first‑isolate rule. KPI‑2: ≥80% lab–drug pairs meet minimum n. KPI‑3: National M39‑compliant report issued annually. | Annual; site directors & NRL QC unit. | Heterogeneous AST completion and non‑standard antibiograms in baseline data. | [3] |
| 4 | QC/EQA transparency KPI: publish EQA participation and pass‑rate by lab (≥2 rounds/year; ≥80% pass), leveraging regional schemes. | KPI‑1: All sites participate in ≥2 EQA rounds/year. KPI‑2: ≥80% pass rate. KPI‑3: Corrective‑action plans closed ≤60 days. | Biannual; NRL QC unit. | Observed inter‑laboratory variability suggests QA gaps. | [4] |
| 5 | Early‑warning analytics for S. Typhi: automate weekly aberration detection using Farrington/Noufaily models on lab‑adjusted counts; define an investigation SLA. | KPI‑1: Automated models run ≥95% of weeks. KPI‑2: Alerts investigated within 5 business days (SLA ≥90%). | Weekly; NRL epidemiology unit. | Year‑to‑year spikes consistent with potential outbreak signals need systematic detection. | [5] |
| 6 | Turn‑around‑time (TAT) KPI for blood culture: track median and 90th‑percentile sample→AST‑report TAT as a process metric. | KPI‑1: Median TAT ≤72 h. KPI‑2: 90th percentile TAT ≤120 h. KPI‑3: ≥95% completeness of time‑stamps in LIS/WHONET. | Monthly; site labs with NRL oversight. | Process delays limit clinical utility of AMR data. | [6] |
| 7 | Genome‑informed policy loop: sequence a quarterly, epidemiologically selected panel; analyze in Pathogenwatch and publish lineage/AMR summaries via TyphiNET to inform empiric therapy and outbreak control. | KPI‑1: Sequence ≥10–20% of national Typhi/Paratyphi isolates per quarter. KPI‑2: ≤60 days from collection to genomic report. KPI‑3: 100% quarterly uploads to Pathogenwatch/TyphiNET. | Quarterly, NRL genomics team. | Bridges phenotype AMR to lineage dynamics; enables cross‑border comparability. | [7] |
| 8 | One‑Health panel harmonization: align human/veterinary core AST panels and data fields; integrate dashboards and joint reviews. | KPI‑1: ≥80% animal isolates tested on a human‑matched core panel within 12 months. KPI‑2: ≥3 joint human–animal AMR review meetings/year. KPI‑3: Integrated dashboard online. | Quarterly, NRL with veterinary reference laboratory. | Supports source‑tracing for high FQ/TMP‑SMX resistance and food‑water pathways. | [8] |

**References**

[1] WHO. Global antimicrobial resistance and use surveillance report (GLASS). ISBN: 9789240062702. <https://www.who.int/publications/i/item/9789240062702>

[2] WHO publication. ISBN: 9789241512411. <https://www.who.int/publications/i/item/9789241512411>

[3] CLSI 2020. Analysis and Presentation of Cumulative Antimicrobial Susceptibility Test Data (M39). <https://www.nih.org.pk/wp-content/uploads/2021/02/CLSI-2020.pdf>

[4] ASLM. EQuAFRICA external quality assessment programme. <https://old.aslm.org/what-we-do/amr/equafrica/>

[5] Early-warning analytics for communicable diseases (Farrington/Noufaily methods). <https://pmc.ncbi.nlm.nih.gov/articles/PMC10576884/>

[6] WHO GLASS—timeliness/quality emphasis <https://www.who.int/publications/i/item/9789240062702>

[7] Pathogenwatch/TyphiNET public‑health genomics resource. PubMed ID: 40346656. <https://pubmed.ncbi.nlm.nih.gov/40346656/>

[8] Africa CDC. New guidance to strengthen AMR surveillance. <https://africacdc.org/news-item/new-guidance-sets-path-to-strengthen-amr-surveillance/>
